# *Staphylococcus aureus*-induced proteomic changes in the mammary tissue of rats: A TMT-based study

**DOI:** 10.1371/journal.pone.0231168

**Published:** 2020-05-04

**Authors:** Lirong Cai, Jinjin Tong, Zhaonan Zhang, Yonghong Zhang, Linshu Jiang, Xiaolin Hou, Hua Zhang

**Affiliations:** 1 Beijing Key Laboratory of Dairy Cow Nutrition, Animal Science and Technology College, Beijing University of Agriculture, Beijing, China; 2 Beijing Key Laboratory of Traditional Chinese Veterinary Medicine, Animal Science and Technology College, Beijing University of Agriculture, Beijing, China; University of Illinois, UNITED STATES

## Abstract

*Staphylococcus aureus* is one of the most important pathogens causing mastitis in dairy cows. The objective of this study was to establish a rat model of mastitis induced by *S*. *aureus* infection and to explore changes in the proteomes of mammary tissue in different udder states, providing a better understanding of the host immune response to *S*. *aureus* mastitis. On day 3 post-partum, 6 rats were randomly divided into two groups (n = 3), with either 100 μL of PBS (blank group) or a *S*. *aureus* suspension containing 2×10^7^ CFU·mL^−1^ (challenge group) infused into the mammary gland duct. After 24 h of infection, the rats were sacrificed, and mammary gland tissue was collected. Tandem mass tag (TMT)-based technology was applied to compare the proteomes of healthy and mastitic mammary tissues. Compared with the control group, the challenge group had 555 proteins with significant differences in expression, of which 428 were significantly upregulated (FC>1.2 and *p*<0.05) and 127 were downregulated (FC>0.83 and *p*<0.05 or *p*<0.01). Gene Ontology (GO) and Kyoto Encyclopedia of Genes and Genomes (KEGG) enrichment analyses revealed that upregulated differentially significant expressed proteins (DSEPs) were associated with mainly immune responses, including integrin alpha M, inter-α-trypsin inhibitor heavy chain 4, and alpha-2-macroglobulin. This study is the first in which a rat model of *S*. *aureus-*induced mastitis was used to explore the proteins related to mastitis in dairy cows by TMT technology, providing a model for replication of dairy cow *S*. *aureus*-induced mastitis experiments.

## Introduction

Mastitis is a highly prevalent disease in dairy cows[1], causing great economic loss and severely restricting the development of the dairy cow breeding industry. *Staphylococcus aureus* (*S*. *aureus*) is one of the common etiological agents of contagious bovine mastitis[2]and is widely distributed in several countries[3]. Previous studies have shown that *S*. *aureus* can survive and cause damage in bovine mammary epithelial cells[4], resulting in contamination of milk with its toxins[5], as well as affecting milk production[6] and triggering a complex host immune response that involves immunity. Furthermore, *S*. *aureus*-induced mastitis is resistant and prone to persistent or recurrent infections[7]. However, due to the particularity of the mammary gland position and physiological structure of dairy cows, as well as the increase in drug-resistant strains, understanding the mechanism of the host response to *S*. *aureus* infection is important for the development of innovative strategies for prevention or treatment of mastitis.

Currently, with the rapid development of technologies such as genomics, transcriptomics and proteomics, high-throughput profiling of identified differentially significant expressed proteins (DSEPs) is associated with abundant information on many diseases as a powerful tool for exploring the underlying mechanism. Proteomics has been widely used to investigate proteomic changes, and potential biomarkers have been identified in the mammary tissue[8], milk[9] and serum[2] of dairy cows with different mammary gland health statuses. However, few proteomics studies have investigated the effects of *S*. *aureus*-induced mastitis in rats. Here, we present an exploration of *S*. *aureus*-induced changes in the mammary gland of rats. Quantitative tandem mass tag (TMT) proteomics is an attractive source of biological information, especially in the context of identifying biomarker proteins. Proteomic analysis will improve our global view of the molecular mechanism involved in *S*. *aureus*-induced mastitis and will guide further studies designed to investigate the pathogenesis of *S*. *aureus* related to the immune system and inflammation during the onset of mastitis as well as provide a model for the replication of dairy cow *S*. *aureus* mastitis experiments.

## Materials and methods

### Animals and tissue collection

The experiments were performed in accordance with the Guide for the Care and Use of Laboratory Animals of the National Research Council. The experimental protocol was approved by the animal care and use committee of Beijing University of Agriculture, China (BUAEC 2018–0205). The study was carried out on pregnant Sprague-Dawley (SD) rats, which were purchased from Beijing Vital River Laboratory Animal Technology Co., Ltd. The *S*. *aureus* strain (ATCC29740) known as Newbould 305 was originally isolated from a cow with mastitis and has been widely used as a model strain able to reproducibly induce chronic mastitis in cows[10–14]; the strain was purchased from the China General Microbiological Culture Collection Center (CGMCC).

*S*. *aureus* was incubated on solid Luria-Bertani (LB, Invitrogen, USA) broth medium for 14–16 h at 37°C. A single colony was transferred to 15 mL of liquid LB (Gibco, USA) medium and cultured for 4 h in a shaker (37°C, 240 r/min). The colony-forming units (CFU) were calculated by the serial dilution plating method. The bacterial solution was diluted to 2×10^7^ CFU·mL^-1^ with endotoxin-free phosphate-buffered saline (PBS, Gibco, USA) before use[15,16].

Six pregnant rats of the same age weighing 265 ± 15 g were raised in plastic cages with sterilized saw dust and fed complete feed at a constant temperature (25°C) and constant humidity, with free access to feed and water until delivery. The rats were randomly divided into two experimental groups, namely, an experimental challenge group (n = 3 rats challenged with *S*. *aureus*) and an experimental blank group (n = 3 rats receiving only the excipient infused into the gland), which was based on the method of Wang[16], 3 days postpartum. Then, either the *S*. *aureus* suspension containing 2×10^7^ CFU·mL^−1^ (challenge group) or PBS (blank group) was injected into the fourth pair of mammary gland tissues through a papillary tube (both sides). Clinical signs such as appetite and mental state were observed and recorded from 0 h to 24 h.

According to the methods of Zhang[17] and Suzuki-Hatano[18], all the rats were euthanized by cervical dislocation after anesthetization by 2% pentobarbital sodium injection (all rat pups were euthanized by this method) at 24 h, and mammary gland tissue was obtained under aseptic conditions. Mammary tissues were weighed, ground homogeneously with PBS (W:V = 1:10) in an ice bath, and then centrifuged at 10,000 r/min for 5 min at low temperature (4°C). The supernatant was discarded, and the sediment was suspended in PBS and mixed evenly. Bacterial CFU were counted by the serial dilution plating method, and the level of infection was estimated as CFU per gram of mammary tissue[19]. To ensure successful induction of the rat mastitis model, another part of the mammary glands was fixed with 10% formaldehyde to make hematoxylin-eosin (H&E)-stained sections for histological examination. Mammary gland tissues after 24 h of infection were pretreated with liquid nitrogen for 15 min, stored at -80°C and used for TMT quantitative proteomic detection and differential protein expression analysis.

### Protein extraction and quantification

All samples were obtained and weighed, and 0.2 g was ground to powder in an ice bath. An appropriate amount of protein lysis buffer (8 M urea+1% sodium dodecyl sulfate (SDS), containing protease inhibitor) was added to lyse the samples on ice for 30 min, with vortex mixing for 5–10 s every 5 min. Ultrasonic pulses were applied on ice for 2 min, and after centrifugation at 12,000 ×g at 4°C for 30 min, the supernatant was obtained. According to the operation method of the Thermo Scientific Pierce BCA Kit, standard protein solutions with concentrations of 0, 0.125, 0.25, 0.5, 0.75, 1, 1.5, and 2 μg μL^-1^ and a BCA working solution were prepared. Taken 2 μL of the sample, 18 μL of distilled water and 200 μL of the BCA working solution were added. Oscillatory mixing was performed, and the samples were reacted at 37°C for 30 min. The absorbance at 562 nm was read with a SPECTRA MAX microplate reader. Then, SDS-PAGE and Coomassie blue staining were used to compare the consistency of protein expression in the samples.

### Reductive alkylation and enzymatic hydrolysis

Taken 100 μg of protein sample, lysis solution was added to bring the volume up to 100 μL, and triethyl ammonium bicarbonate (TEAB) buffer was added at a final concentration of 100 mM. Then, 10 mM reducing agent (Bond-Breaker^™^ TCEP solution) was added, and the reaction time was 60 min at 37°C. Next, 40 mM iodoacetamide was added and reacted at room temperature for 40 min in darkness. Precooled acetone (W:V = 1:6) was added; then, the sample was precipitated at -20°C for 4 h and centrifuged at 12,000 ×g for 20 min, and the precipitate was transferred. The sample was fully dissolved with 100 μL of 50 mM TEAB. Enzymatic hydrolysis was allowed to proceed overnight at 37°C by adding trypsin according to the mass ratio (enzyme:protein = 1:50).

The TMT reagent was centrifuged, and after acetonitrile was added and vortex mixing was performed, a tube of TMT reagent was added to each 100 μg of polypeptide. The samples were labeled as follows: Blank-1 and Blank-2, TMT10-127C; Blank-3 and Challenge-1, TMT10-129C; Challenge-2 and Challenge- 3, TMT10-128N. After incubation for 2 h, hydroxylamine was added and reacted at room temperature for 15 min. Equivalent labeled products were mixed in a tube and dried by a vacuum concentrator.

### High-pH reversed-phase liquid chromatography

The peptide samples were redissolved in ultra-performance liquid chromatography (UPLC) buffer solution, and a reversed-phase C18 column (2.1 mm×150 mm, 1.7 μm-C18) was used to separate the components in a high-pH liquid phase. The buffer solutions were as follows: A, 2% acetonitrile; and B, 80% acetonitrile (both adjusted with ammonia to pH = 10). The ultraviolet detection wavelength was 214 nm, and the flow rate was 200 μL/min. The UPLC gradient was as follows: 0~2 min, 0~5% B; 2~17 min, 5% B; 17~35 min, 5%~30% B; 35~38 min, 30%~36% B; 38~39 min, 36%~42% B; 39~40 min, 42%~100% B; 40~45 min, 100%~0 B; and 45~48 min, 0% B. A total of 20 fractions were collected from each group according to peak type and time, combined into 10 fractions, and concentrated by vacuum centrifugation.

### LC-MS/MS analysis

The second dimension used a nanoliter-scale high-performance liquid chromatography (HPLC) system (EASY-nLC 1200) for separation at a 300 μL/min flow rate for 120 min. Peptide segments were dissolved in mass spectrometry sample buffer and prepared in buffer solution (A: 2% acetonitrile, 0.1% formic acid; B: 80% acetonitrile, 1% formic acid). The EASY-nLC liquid phase gradient was as follows: 0~4 min, 0~5% B; 4~66 min, 5%~23% B; 66~80 min, 23%~29% B; 80~89 min, 29%~38% B; 89~91 min, 38~48% B; 91~92 min, 48%~99% B; 92~105 min, 99% B; 105~106 min 99~0% B; and 106~120 min, 0% B. The 20 strongest signals of the parent ion were selected using Q-Exactive Plus (Thermo, USA) for secondary fragmentation (mass spectrometry scanning range, 350–1,300 m/z; acquisition mode, data-dependent acquisition (DDA)). The resolution for primary mass spectrometry was 70,000, the automatic gain control (AGC) target was 3e6, the maximum injection time was 20 ms, and the fragmentation type was high-energy collision dissociation (HCD). The secondary mass spectrometry resolution was 35,000 at 100 m/z, the AGC target was 1e5, and the maximum injection time was 50 ms.

### Statistical analysis

The data were analyzed using SPSS software (version 17.0; SPSS Institute, USA), and the statistical results were expressed as the means ± standard errors. There were three biological replicates in each experimental group.

### Protein mass spectrometry data statistics

The raw files were submitted to the Proteome Manufacturer (Proteome Discoverer^TM^ Software 2.2) server; a database that has been established was selected, and then, a database search was performed. The data are available via ProteomeXchange (http://www.proteomexchange.org) with the identifier PXD017540.

For protein identification, the Paragon algorithm, which was integrated into Protein Pilot, was employed against the SwissProt bovine database from the UniProt website (http://www.UniProt.org) using Mascot software, version 2.3.02 (Matrix Science, London, UK). Differentially significant expressed proteins (DSEPs) were filtered against the standard (fold change≥1.2 and p-value<0.05) based on protein abundance level.

To determine the biological and functional properties of all the identified proteins, the identified protein sequences were mapped with Gene Ontology (GO, http://geneontology.org/) terms. The Clusters of Orthologous Groups of proteins (COG, http://www.ncbi.nlm.nih.gov/COG/) database was employed for functional annotation of genes from new genomes and for research into genome evolution. To identify candidate biomarkers, we employed a hypergeometric test to perform GO enrichment and pathway enrichment. Protein classification was performed based on functional annotations using GO terms for biological process (BP), cell component (CC) and molecular function (MF).

## Results

### Clinical signs and histopathological examination

The rats in the blank group exhibited no abnormal appearance during the observation period. However, the challenge group showed clinical signs consistent with mastitis, including redness, fever and swelling. Compared with the blank group, the challenge group had a poor mental state and exhibited loss of appetite and low activity.

**Fig 1:**
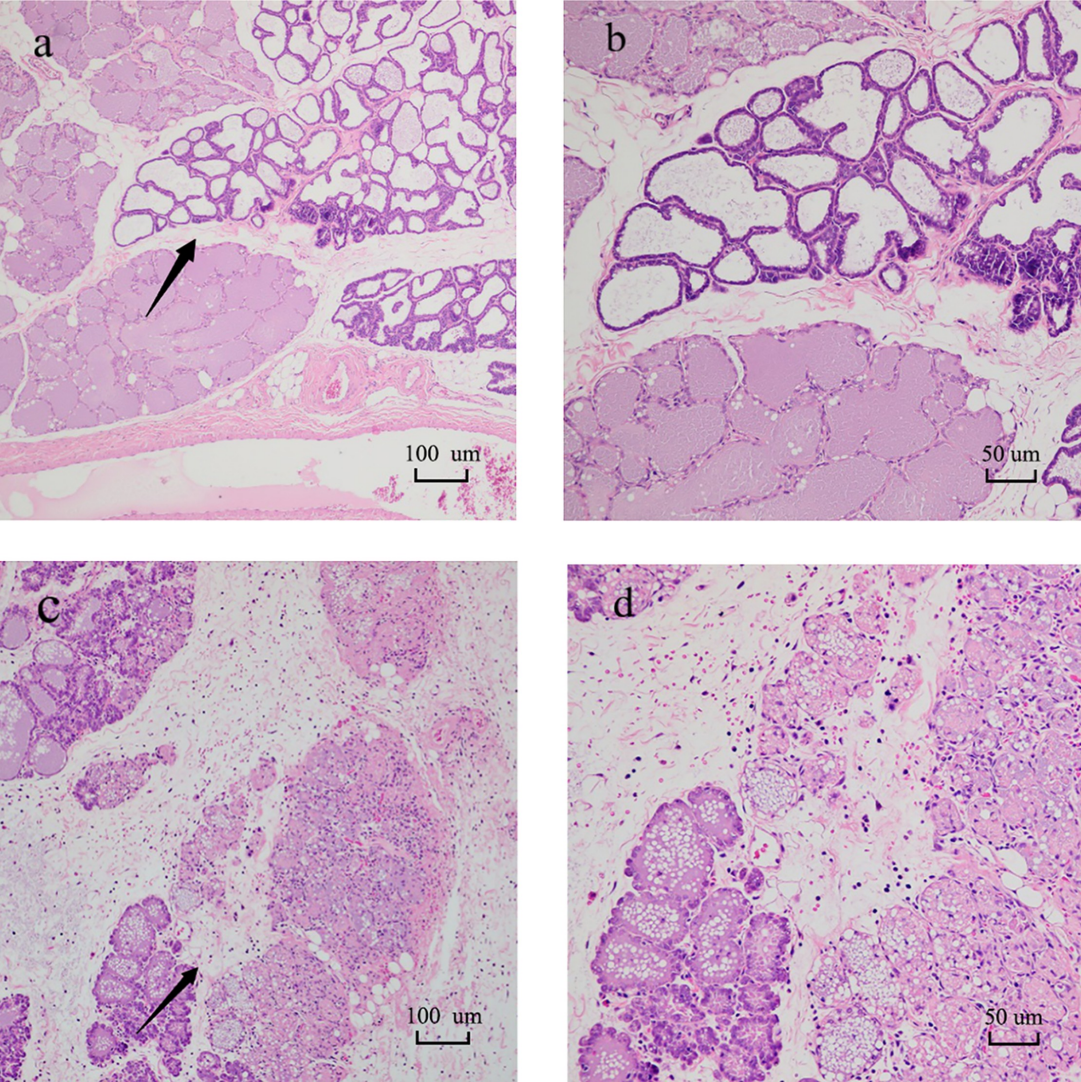
Results of H&E staining in rat mammary tissue for the blank group **(a, b)** and challenge group **(c, d)**. Compared with a, the interlobular space of c was widened, accompanied by significant inflammatory edema and partial acinar atrophy, with acinar epithelial cells undergoing degeneration and necrosis and infiltration of neutrophils, macrophages, and lymphocytes. **b** and **d** (H&E×200) are enlarged images of the region designated by the arrows in **a** and **c** (H&E×100), respectively.

In the blank group, the mammary gland structure was complete, the mesenchyme was thin and uniform, and the acinar cavity was closely arranged, which indicated that there were no significant changes in tissues in the blank group (Fig 1A and 1B). The mammary gland tissue of rats in the challenge group showed severe damage (Fig 1C and 1D), including necrosis of tissue cells, infiltration of inflammatory cells and formation of pyogenic foci.

### Examination of the bacterial count in mammary gland tissue

No bacteria were isolated from the mammary gland tissue of the blank group. At 24 h after *S*. *aureus* injection, the amount of bacteria in the mammary gland tissue of the challenge group was 7.70 ± 0.16 log10 CFU/g.

### Identification and quantitative analysis of proteins

**Fig 2:**
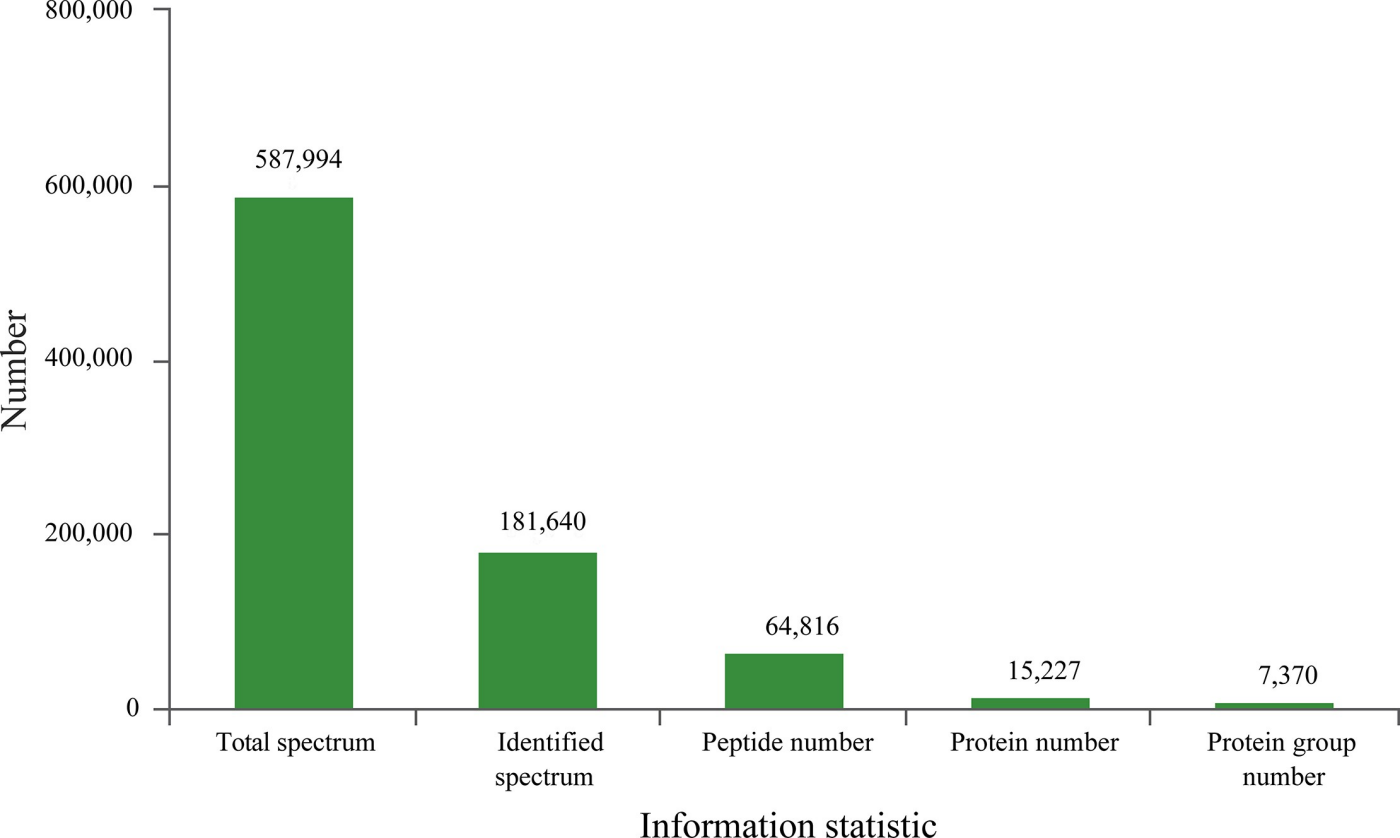
Information analysis for protein identification.

**Fig 3:**
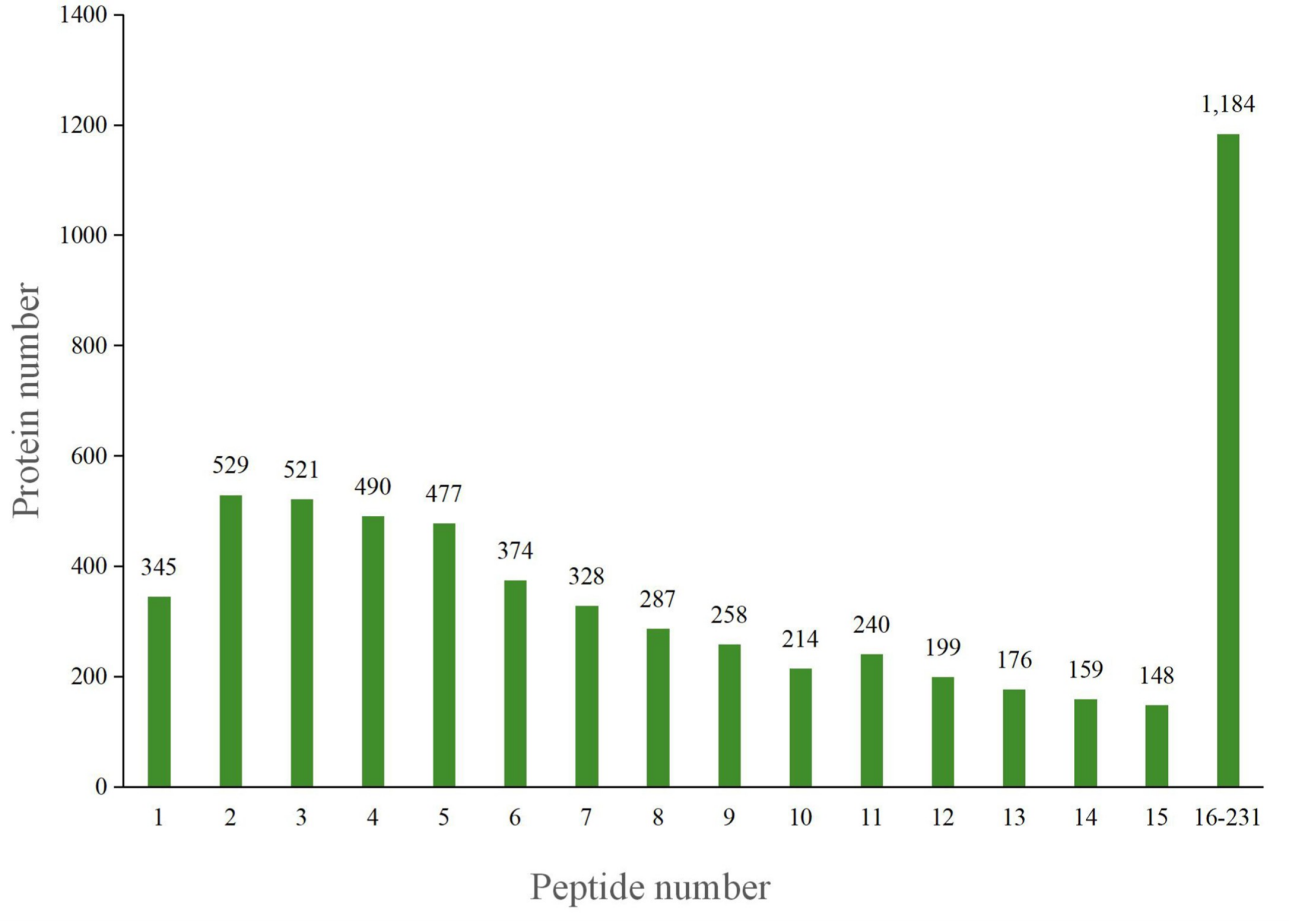
Quantitative distribution of peptides in identified proteins.

**Fig 4:**
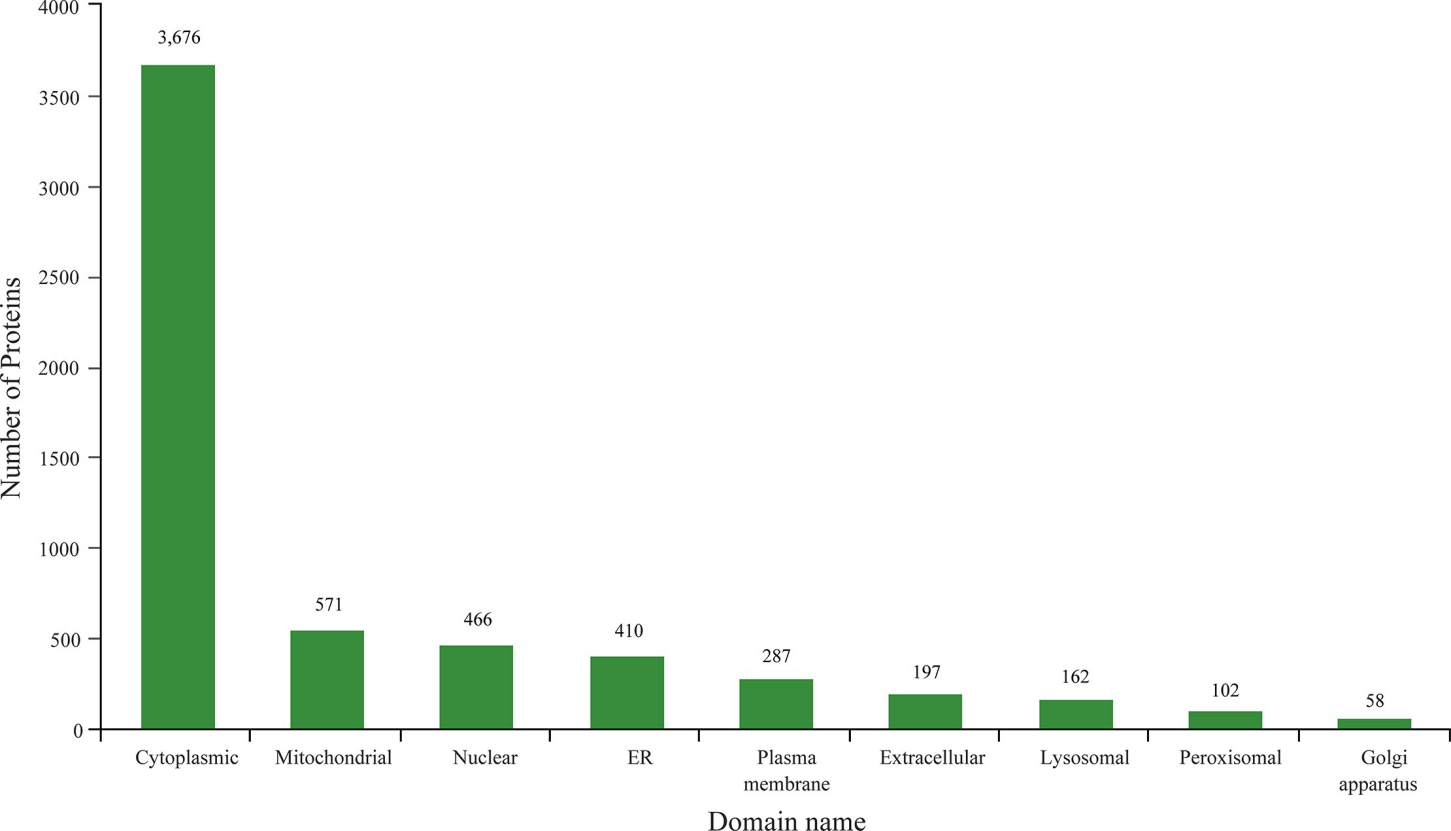
Prediction of protein subcellular localization.

The total spectrum identified consisted of 587,994 species, and the number of matched spectra was 181,640. A total of 64,816 peptide segments and 15,227 proteins were identified. The number of protein groups was 7,370 (Fig 2). After data analysis, among the 5,929 proteins that could be quantified, 5.8% were single peptides, with dipeptides accounting for the maximum proportion, 8.92% (Fig 3). MultiLoc 2 software was used to predict protein subcellular localization, and the results showed that 3,676 proteins were annotated to the cytoplasm, accounting for 62% of the total (Fig 4).

### Identification of DSEPs

**Fig 5:**
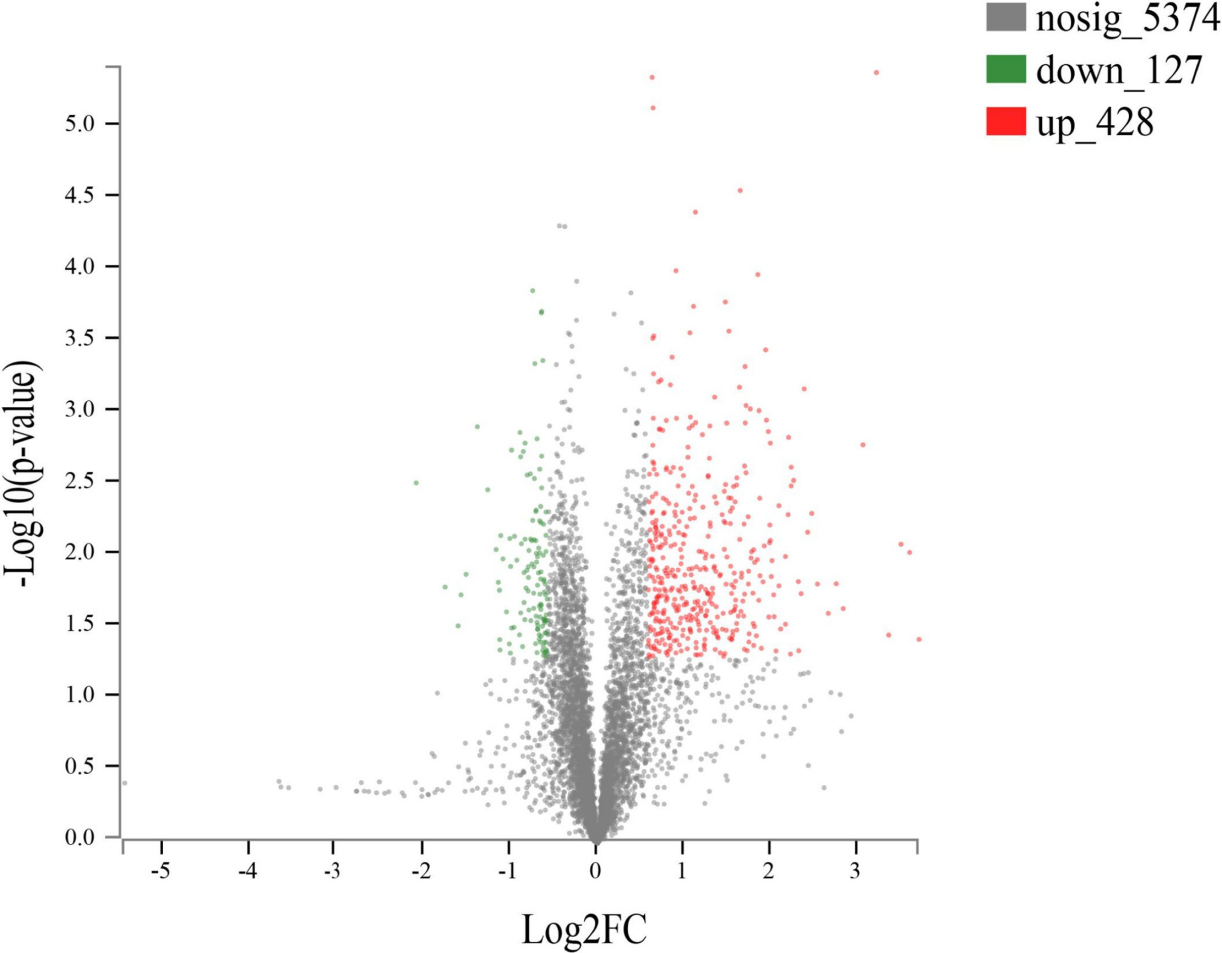
Volcano plot of DSEPs in the challenge group compared to the blank group. Each point in the figure represents a specific protein. The abscissa is the fold change in the difference in protein expression between the two groups; the ordinate represents -log10 (*p*-value). The farther away from zero the point is, the more significant the difference.

As shown in the volcano plot (Fig 5), among the 5,929 proteins that could be quantified, 555 DSEPs were identified in the challenge group compared to the blank group, of which 428 were upregulated and 127 were downregulated (DSEPs were defined as proteins for which the fold change was >1.20 or <0.83 and the *p*-value was <0.05).

### GO functional enrichment analyses of DSEPs

GO (http://www.geneontology.org/) is a database established by the Gene Ontology Association. The GO functions of up- and downregulated DSEPs were annotated, and the BP, CC and MF terms of proteins involved at the functional level were analyzed.

**Fig 6:**
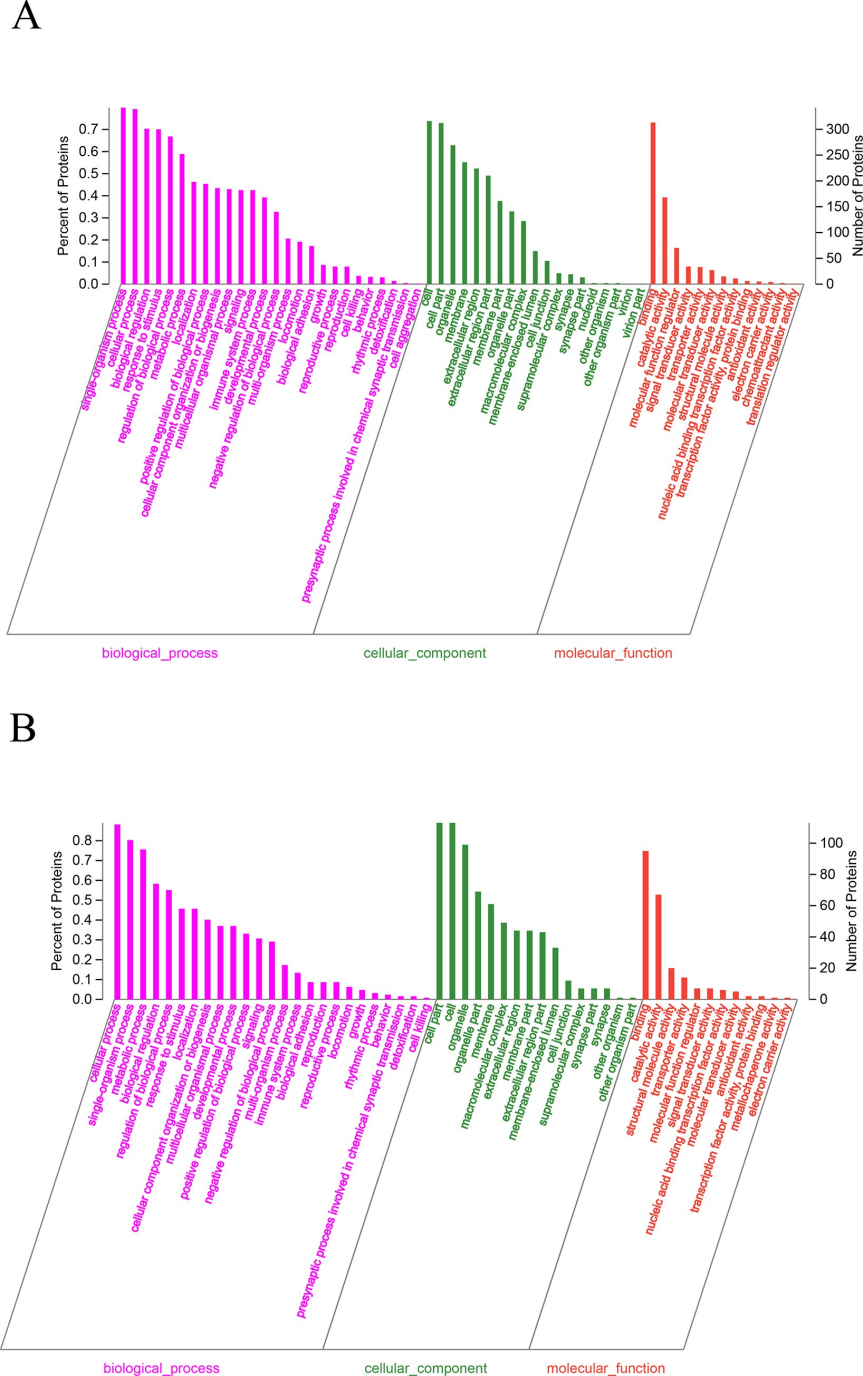
GO enrichment analysis of DSEPs. (**A**) Upregulated DSEPs and (**B**) downregulated DSEPs. The x-axis represents the secondary GO classification term, while the y-axis (left) denotes the percentage of DSEPs included in the secondary classification from the total number of DSEPs in the secondary classification, and the y-axis (right) denotes the number of proteins associated with the secondary classification.

As shown in Fig 6A and 6B, the GO functional enrichment results for the two groups were similar, with most DSEPs annotated in the BP category. Among the upregulated DSEPs, the highest proportion were annotated as single-organism process and cell processes, followed by response to stimulus, biological regulation and regulation of biological process. Among the downregulated DSEPs, cellular process and single-organism process had the greatest number of annotated proteins. In the CC group, most proteins were associated with cell and cell part. Whether upregulated or downregulated, most of the DSEPs were involved in the MF category of binding, and catalytic activity was also found to be enriched with downregulated DSEPs.

### KEGG pathway enrichment analyses of DSEPs

In organisms, different gene products perform their specific biological functions through orderly coordination. Therefore, through KEGG functional enrichment analyses, we can identify the metabolic pathways in which DSEPs are mainly involved. The upregulated DSEPs participated in a total of 41 metabolic pathways (second category), and the 10 metabolic pathways with the greatest number of proteins are listed in Table 1, including mainly the immune system, signal transduction, and cancer: overview. The downregulated DSEPs were involved in only 30 metabolic pathways, and Table 2 shows the top ten pathways with the greatest number of proteins, including global and overview maps, translation and the endocrine system pathway.

**Table 1:** Enrichment analysis of the top ten pathways associated with the upregulated DSEPs.

First Category	Second Category	Protein Number
Organismal Systems	Immune system	82
Environmental Information Processing	Signal transduction	48
Human Diseases	Cancer: Overview	41
Cellular Processes	Transport and catabolism	39
Human Diseases	Infectious diseases: Bacterial	33
Human Diseases	Infectious diseases: Parasitic	27
Human Diseases	Immune diseases	23
Cellular Processes	Cellular community-eukaryotes	21
Human Diseases	Infectious diseases: Viral	21
Organismal Systems	Endocrine system	19

**Table 2:** Enrichment analysis of the top ten pathways associated with the downregulated DSEPs.

First Category	Second Category	Protein Number
Genetic Information Processing	Translation	14
Organismal Systems	Endocrine system	13
Metabolism	Global and overview maps	11
Metabolism	Amino acid metabolism	10
Metabolism	Lipid metabolism	10
Metabolism	Carbohydrate metabolism	7
Human Diseases	Cancer: Overview	7
Cellular Processes	Transport and catabolism	6
Metabolism	Metabolism of other amino acids	5
Environmental Information Processing	Signal transduction	5

**Fig 7:**
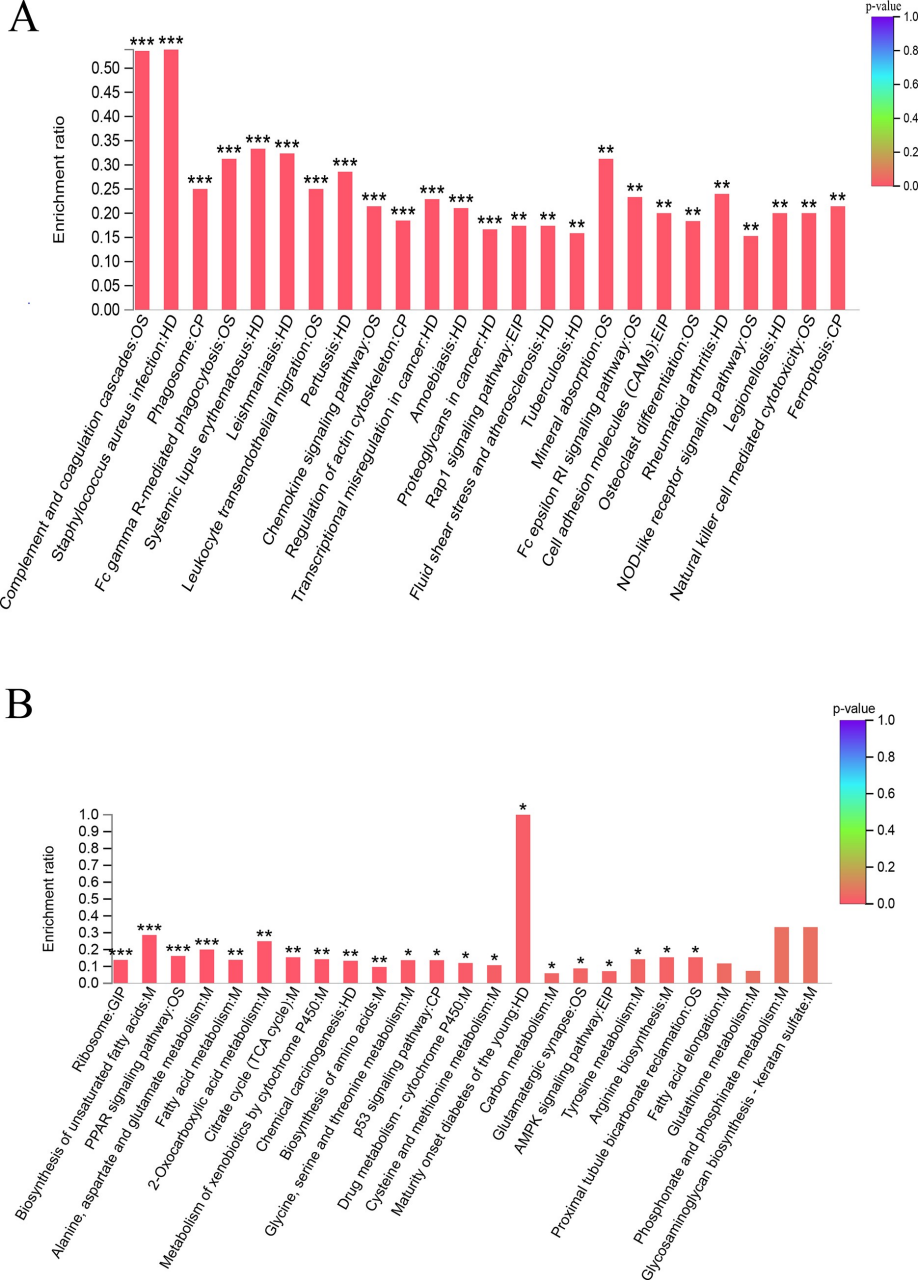
KEGG enrichment analyses of DSEPs. (**A**) Upregulated DSEPs and (**B**) downregulated DSEPs (***p < 0.001, **p < 0.01, *p < 0.05). The x-axis represents the pathway name, while the y-axis denotes the enrichment ratio. M: metabolism; GIP: genetic information processing; EIP: environmental information processing; CP: cellular processes; OS: organismal systems; HD: human diseases.

Using Fisher's exact test and taking all qualitative proteins as the background, when the corrected *p*-value (Padjust) was <0.05, he KEGG function was considered to be significantly enriched. The enrichment rate is the ratio of the number of proteins enriched in the pathway to the number of proteins annotated to the pathway. As shown in Fig 7A and 7B (the top 25 pathways from the KEGG enrichment analysis), the upregulated DSEPs were enriched in 221 pathways (third category), including the complement and coagulation cascades pathway (rno04610) and the *S*. *aureus* infection pathway (rno05150), which had the highest enrichment rate of approximately 54%. The mineral absorption pathway (rno04978), Fc gamma R-mediated phagocytosis pathway (rno04666), and systemic lupus erythematosus pathway (rno05322) also had increased enrichment rates. The downregulated DSEPs were enriched in 111 pathways. P70562 (class A basic helix-loop-helix protein 15) was enriched in the maturity onset diabetes of the young pathway (rno04950) with only one background protein, and the enrichment rate was 100%. The biosynthesis of unsaturated fatty acids pathway (rno01040), phosphonate and phosphinate metabolism pathway (rno00440) and glycosaminoglycan biosynthesis-keratan sulfate pathway (rno00533), with high enrichment rates, all belong to metabolism.

## Discussion

In this study, we hypothesized that *S*. *aureus*-induced mastitis in rats may involve biomarker proteins that provide a global view of the molecular mechanism in dairy cows. This hypothesis was supported by the results; we identified and quantitated the proteins in mammary gland tissue by quantitative TMT proteomics on the basis of the successful establishment of a rat mastitis model, followed by DSEP analysis. Similar to the findings in other microorganisms, *S*. *aureus* infection-induced mastitis is refractory to treatment[20]. The results of GO and KEGG analyses identified DSEPs related to inflammation and immunity during the onset of mastitis, providing a theoretical basis for the pathogenesis of *S*. *aureus*-induced bovine mastitis.

According to the results of GO functional enrichment, the BP category accounts for approximately half of the total functions. The GO secondary classification that annotated additional DSEPs included single-organism process, biological regulation, cellular process and response to stimulus. Among them, the upregulated DSEPs mainly included neutrophil gelatinase-associated lipocalin (LOC100909700, FC = 7.123153), myeloperoxidase (Mpo, FC = 6.73365), and lysozyme (Lyz-2, FC = 5.085199), and the downregulated DSEPs included Muc1 protein (Muc1, FC = 0.499798), cyclin-dependent kinase1 (Cdk1, FC = 0.416559), and parathymosin (Ptms, FC = 0. 565501).

In the present study, mammary tissues from *S*. *aureus*-infected rats lasted only 24 h, during which time, the host's defense against *S*. *aureus* was mainly regulated by the innate immune response. Through innate immune recognition, inflammatory cascades, including the aggregation of leukocytes to the site of infection, induction of the adaptive immune response and activation of antimicrobial mechanisms, were initiated[21]. These signaling pathways are coordinated through complex mechanisms. The results of the functional enrichment of KEGG pathways in this experiment showed that 82 of the upregulated DSEPs participated in the immune system pathway, involving the complement and coagulation cascades pathway (rno04610), Fc gamma R-mediated phagocytosis pathway (rno04666) and leukocyte transendothelial migration pathway (rno04670).

DSEPs that participate in the complement and coagulation cascade pathways account for the largest proportion, that is, the organism may first regulate this pathway to fight *S*. *aureus* infection. Thirty upregulated DSEPs, such as coagulation factor (X, VII), complement component (C3, C9) and alpha-2-macroglobulin (A2M), participate in the complement and coagulation cascade pathways. This pathway belongs to the innate immune system and shares numerous interactions with components of the hemostatic pathway[22]. C5 and C9 participate in the complement activation pathway, and the complement system is a main line of defense against infection[23]. It was reported that A2M can affect its function by binding with TNF-α[24], IL-6[25] and other cytokines and plays a role in balancing inflammation and anti-inflammation.

With the development of mastitis, the pathways related to the migration of inflammatory cells were significantly regulated, which caused inflammatory cells and factors to aggregate in the lesion to produce comprehensive proinflammatory and anti-inflammatory effects. The H&E staining results showed that inflammatory cells, such as neutrophils and macrophages, were concentrated in the lumen during 24 h of mammary tissue challenge by *S*. *aureus*. Integrin (Itg) participates in proliferation, migration[26] and signal transduction[27]. Itg can mediate the adhesion of neutrophils to vascular endothelial cells[28], induce proinflammatory cytokine genes[27], and participate in physical defense. In this experiment, both integrin beta (Itgb2), integrin alpha M (Itgam) and integrin subunit alpha L (Itgal) were found to be upregulated. These proteins participate in multiple pathways, including complement and coagulation cascades, phagosome, and the *S*. *aureus* infection pathway. Vasodilator-stimulated phosphoprotein (Vasp) is an actin-binding protein that regulates leukocyte aggregation and polarization[29]. Vasp, neutrophil cytosolic factor 4 (Ncf4), rac family small GTPase 2 (Rac2), etc., participate in the leukocyte transendothelial migration (rno04670) pathway.

Immune cells can cause damage to the blood-milk barrier during migration[8]. Upregulation of fibronectin (Fn1), actin-related protein 2/3 complex subunit 1B (Arpc1b), vitronectin (Vtn) and other related proteins plays an important regulatory role. Moreover, these DSEPs are involved in the regulation of actin cytoskeleton (rno04810), focal adhesion (rno04510) and ECM-receptor interaction (rno04512) pathways, which are essential for maintaining cell and tissue structure. Therefore, in the model of *S*. *aureus*-induced mastitis in rats, protein expression in these pathways is upregulated to fight against bacteria and reduce the cell and tissue damage caused by infection.

In addition, stimulation of the Fc gamma R-mediated phagocytosis pathway causes actin cytoskeleton polymerization and affects phagocytosis. Fifteen DSEPs, including cytochrome b-245 beta chain (Cybb), protein kinase C delta type (Prkcd) and 1-phosphatidylinositol 4,5-bisphosphate phosphodiesterase (Plcb4), are involved in this pathway. High expression of thrombospondin 1 (Thbs 1) was also found in this study. Thbs 1 is an extracellular matrix glycoprotein that plays a significant role in regulating cell adhesion and death, cell migration, and apoptosis[30]; this protein is involved in the phagosome pathway (rno04145). Phagosomes are essential for reducing the spread of intracellular pathogens[31]. Moreover, 14 DSEPs, such as C3, low-affinity immunoglobulin γ Fc region receptor III-like and plasminogen, were enriched in the *S*. *aureus* infection pathway.

Acute-phase reactions occur 12 to 48 h after the body is stimulated by inflammation. During this period, the serum amyloid A, haptoglobin (Hp), alpha-1-acid glycoprotein (Orm1), alpha-1-antitrypsin and inter-α-trypsin inhibitor heavy chain 4 (ITIH4) protein levels increase in different inflammatory processes. In this study, compared with the blank group, the challenge group exhibited significantly upregulated expression of Orm1, Hp and ITIH4 in the mammary gland. In previous studies, ITIH4 was detected in mammary tissue[32,33], serum[34] and milk[35] of dairy cows with mastitis, and the concentration of this protein in these cows was significantly higher than that in healthy dairy cows. ITIH4 is a glycoprotein produced mainly by the liver and secreted into plasma, belonging to the inter alpha trypsin inhibitor (ITI) family[36]. The molecular weight of this protein is 120 kDa[37], and the protein is likely processed to produce fragments of different lengths; the 100-kDa fragment of bovine ITIH4 plays an important role in the defense and immune responses to mastitis infection[33].

In summary, TMT analyses elucidated the role of A2M, Itgb2, Thbs1, Fn1, ITIH4 and other proteins in the host innate response to *S*. *aureus*-induced mastitis. The DSEPs are involved in pathways such as complement and coagulation cascades, leukocyte transendothelial migration, phagosome pathways and ECM-receptor interaction, which are key to resisting *S*. *aureus* infection-induced mastitis.

## Conclusion

This is the first time that TMT has been used to analyze the mammary tissue of a rat model of *S*. *aureus-induced* mastitis. In this study, we identified the proteins in the mammary tissue, and the immune-related DSEPs were analyzed. DSEPs, such as ITIH4 and A2M, were detected in the mammary tissue of mastitis model rats in this experiment, which demonstrates that the rat model can help elucidate the pathogenesis of *S*. *aureus*-induced mastitis. This study provides a model that replicates mastitis caused by *S*. *aureus* in dairy cows, and these results can help us better understand the molecular mechanism of early infection.

## Supporting information

S1 Checklist The arrive guidelines checklist. (DOCX)
