# Supplementary material for: Staphylococcus aureus-induced proteomic changes in the mammary tissue of rats: A TMT-based study
Source: PLoS One. 2020 May 4;15(5):e0231168. doi: 10.1371/journal.pone.0231168 (PMC7197811; doi:10.1371/journal.pone.0231168)

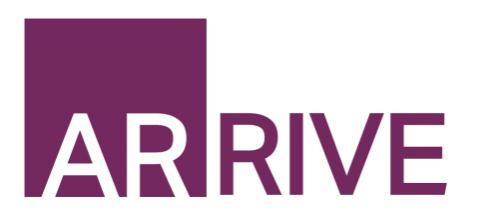


The ARRIVE Guidelines Checklist

Animal Research: Reporting In Vivo Experiments

Carol Kilkenny^1^, William J Browne^2^, Innes C Cuthill^3^, Michael Emerson^4^ and Douglas G Altman^5^

*^1^The National Centre for the Replacement, Refinement and Reduction of Animals in Research, London, UK, ^2^School of Veterinary Science, University of Bristol, Bristol, UK, ^3^School of Biological Sciences, University of Bristol, Bristol, UK, ^4^National Heart and Lung Institute, Imperial College London, UK, ^5^Centre for Statistics in Medicine, University of Oxford, Oxford, UK.*

|  | | ITEM | RECOMMENDATION | Section/ Paragraph |
| --- | --- | --- | --- | --- |
| 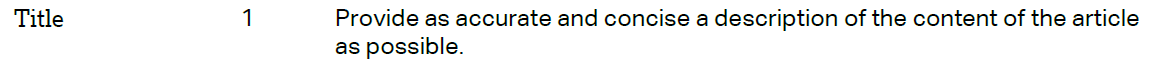 | | | Title |  |
| 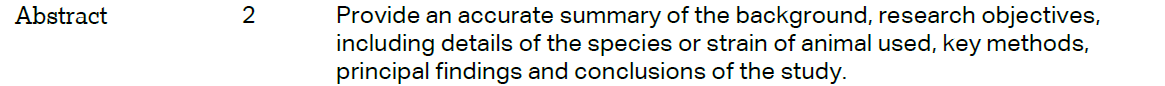 | | | Abstract |  |
| INTRODUCTION | | |  |  |
| 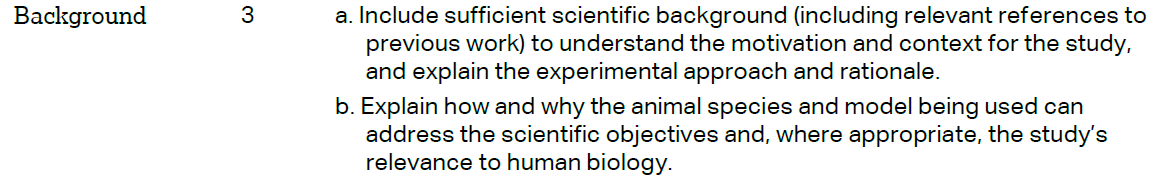 | | | Background，paragraph 1 and 2 |  |
| 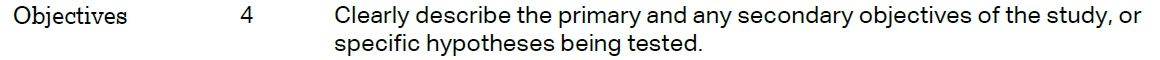 | | | N/A |  |
| METHODS | | |  |  |
| 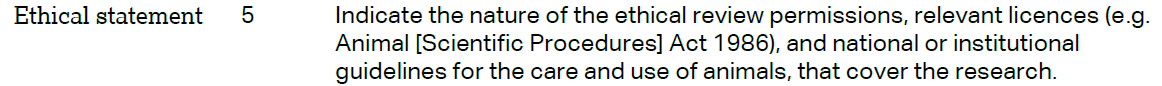 | | | Materials and methods,Animals and Tissue Collection,paragraph 1 |  |
| 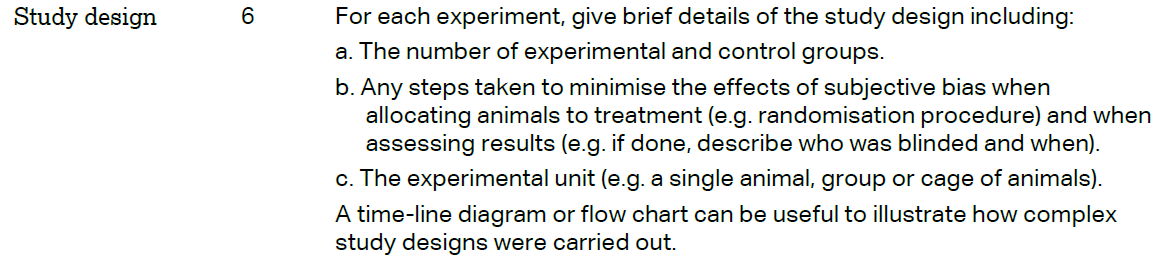 | | | N/A |  |
| 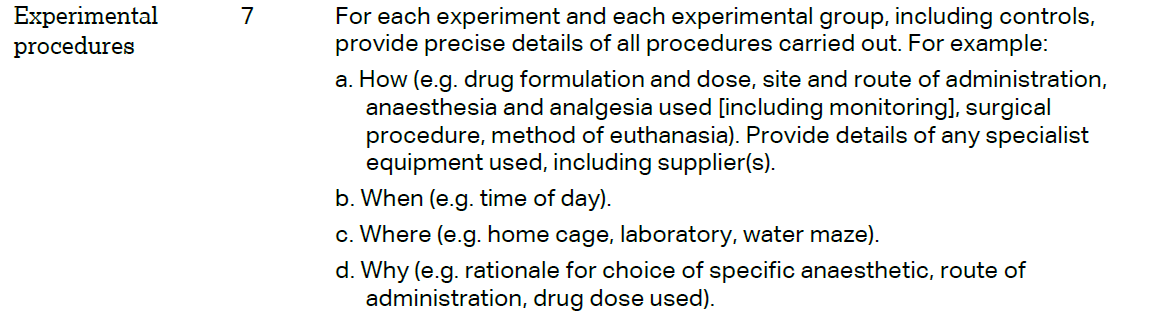 | | | Materials and methods,Animals and Tissue Collection,paragraph 1 and 2 |  |
| 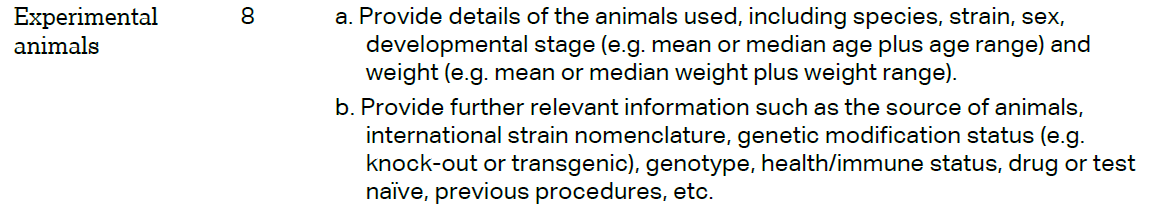 | | | Materials and methods,Animals and Tissue Collection,paragraph 1 |  |

The ARRIVE guidelines. Originally published in *PLoS Biology*, June 2010^1^

| 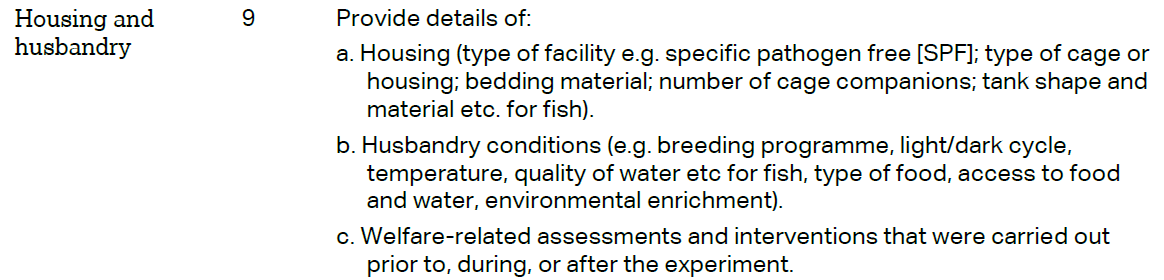 | Materials and methods,Animals and Tissue Collection,paragraph 1 | |
| --- | --- | --- |
| 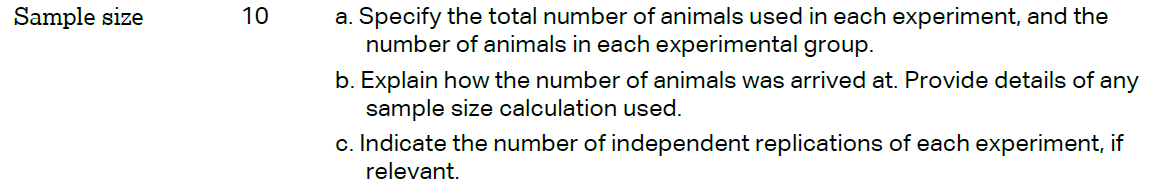 | Materials and methods,Animals and Tissue Collection,paragraph 1 | |
| 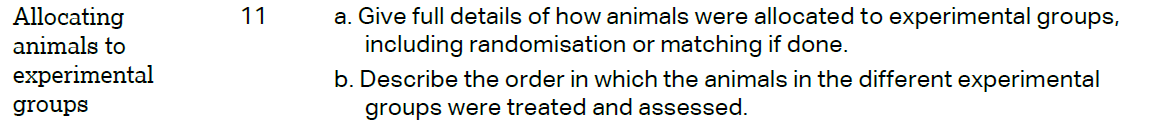 | Materials and methods,Animals and Tissue Collection,paragraph 1 | |
| 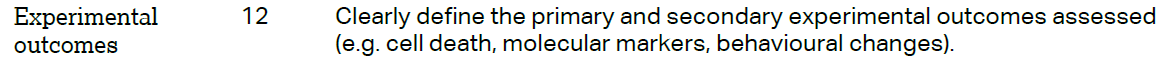 | Materials and methods,Animals and Tissue Collection,paragraph 1 and 2 | |
| 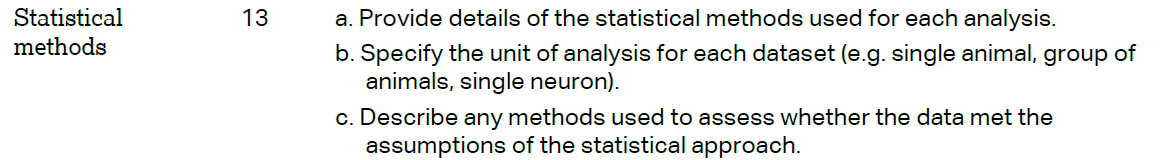 | Materials and methods,Statistics Analysis | |
| RESULTS |  | |
| 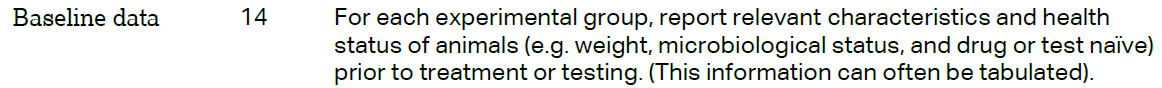 | Results,Establishment of Rat Mastitis Model and Examination of Bacterial Count in Mammary Gland Tissue | |
| 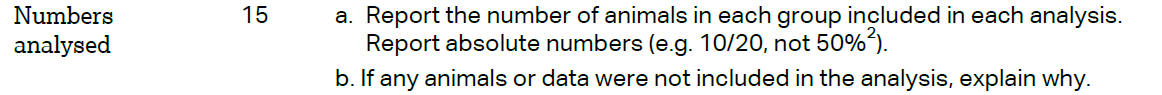 | N/A | |
| 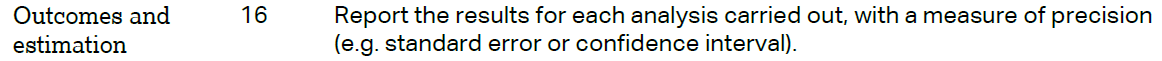 | N/A | |
| 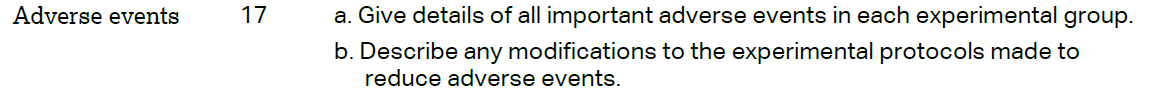 | N/A | |
| DISCUSSION |  | |
| 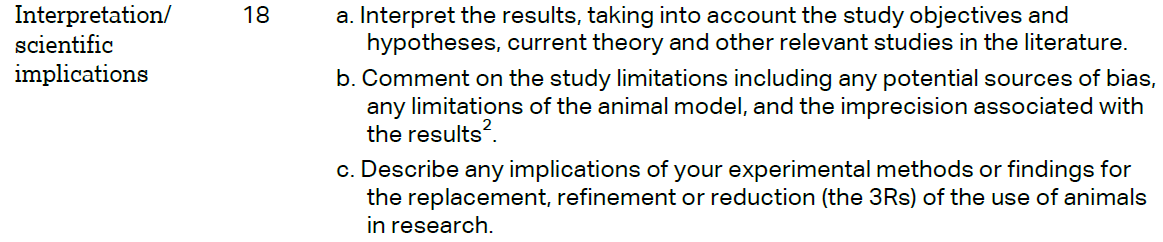 | Discussion,paragraph 1 | |
| 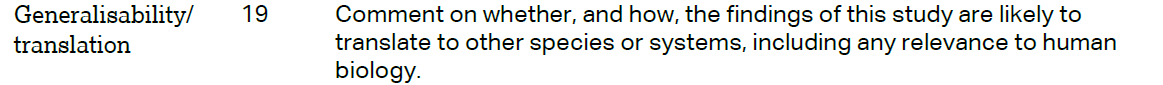 | N/A | |
| 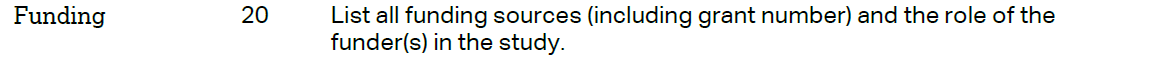 | | N/A |


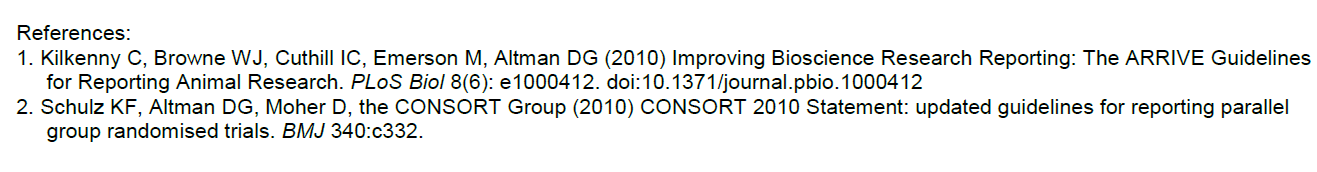

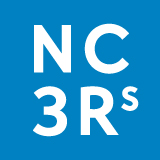

Supplement: S1 Checklist — (DOCX) [file pone.0231168.s001.docx]
